# Automation of oxygen titration in patients with acute respiratory distress at the emergency department. A multicentric international randomised controlled study

**DOI:** 10.1186/2197-425X-3-S1-A424

**Published:** 2015-10-01

**Authors:** E L'Her, P Dias, M Gouillou, N Paleiron, P Archambault, P-A Bouchard, F Lellouche

**Affiliations:** CHRU Brest, Brest, France; CHRU Brest, CIC INSERM 1412, Brest, France; HIA Clermont-Tonnerre, Brest, France; Hôtel Dieu, Lévis, Canada; Institut Universitaire de Cardiologie et de Pneumologie de Québec, Québec, Canada; Institut Universitaire de Cardiologie et de Pneumologie de Québec, Lac-Beauport, Canada

## Introduction

Oxygen therapy is commonly administered in critical care and emergency medicine. Its benefits are well known but potential side-effects may be underestimated. Compliance to recommendations remains dependent on staff workload. We developed FreeO_2_, an innovative device that automatically titrates oxygen flow delivered through nasal cannulas or masks to maintain the patients in the SpO_2_ target set by the clinician^1^.

## Objectives

To compare FreeO_2_ with oxygen manual adjustment, in patient admitted to the emergency department (ED) for acute respiratory failure (ARF).

## Methods

We conducted a multicentre randomized controlled study. Inclusion criteria were: admission to ED for ARF requiring O_2_≥3L/min. Main exclusion criteria were: O_2_≥15 L/min, immediate need for ventilatory support. After inclusion, patients were randomized to either FreeO_2_ or conventional O_2_ manual adjustment during 3 hours. The randomization was web-based and stratified for the type of respiratory failure (hypoxemia/hypercapnia) and for the centre. Primary outcome was the % of time with SpO_2_ within the predefined target (92-96% for hypoxemic patients; 88-92% for hypercapnic patients). Secondary endpoints were: frequency of severe hypoxemia (SpO_2_ < 85%) and hyperoxia (SpO_2_> 98%), partial or complete oxygen weaning at the ED, total O_2_ duration, ventilator support use, ICU admissions, ICU and hospital LOS.

## Results

187 patients were randomized (93 FreeO_2_ and 94 Manual). Baseline physiological characteristics were similar in the 2 groups: age = 76 ± 12 yrs., 35% of COPD patients; mean O_2_ flow at inclusion was 5.8 ± 3.1 L/min. No serious adverse events related to the protocol or device was recorded. The percentage of time within the SpO_2_ target was 81 ± 21% in the FreeO_2_ arm and 51 ± 30% in the Manual arm (P < 0.001). Percentage of time with severe hypoxemia and with hyperoxia were significantly lower with FreeO_2_ (P < 0.001). The percentage of patients with O_2_ flow reduction of more than 50% during the 3 hours of the study was 39% with FreeO_2_ vs 19% in the Manual arm (P = 0.011). Percentage of O_2_ weaning at the end of the study (3h) was 4.3% in the FreeO_2_ arm vs. 14.1 % in the Manual arm; p < 0.001. Significantly less patients in the hypercapnic subgroup were transferred in the ICU, overall O_2_ administration and hospital LOS were significantly reduced in the non-adjusted analysis (9.2 ± 6.9 vs 11.1 ± 7.0, P = 0.01).

## Conclusions

The automation of oxygen therapy with FreeO_2_ at the ED improves the oxygenation parameters with more time in the specified SpO_2_ target, less desaturation and less hyperoxia. FreeO_2_ may reduce staff workload and improve the compliance to recommendations for oxygen administration with potential related clinical benefits

## Grant Acknowledgment

*PHRC National 2010 FreeO2 Hypox #08-12*

*Grant PSR-SIIRI-MDEIE (Ministry of Finances, Québec)*
